# Supplementary material for: Integrative analysis of genomic and transcriptomic data informs precancer progression in the pancreas
Source: bioRxiv. 2025 Nov 4:2025.11.03.686234. Preprint. [Version 1] doi: 10.1101/2025.11.03.686234 (PMC12637499; doi:10.1101/2025.11.03.686234)

bioRxiv preprint doi: https://doi.org/10.1101/2025.11.03.686234; this version posted November 4, 2025. The copyright holder for this preprint (which was not certified by peer review) is the author/funder, who has granted bioRxiv a license to display the preprint in perpetuity. It is made available under aCC-BY 4.0 International license.

Supplementary Figure 1. CoGAPS optimization

A Mean chi squared statistic per CoGAPS run

B Correlation of CoGAPS pattern weights

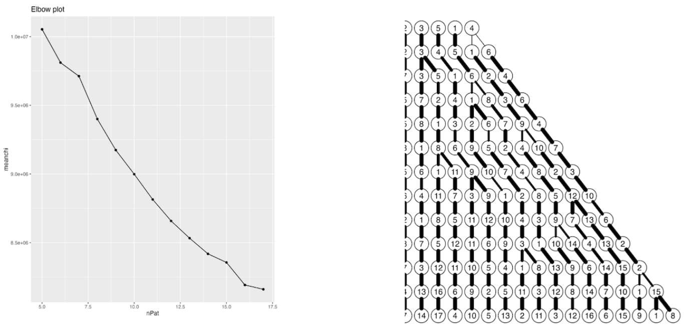

bioRxiv preprint doi: https://doi.org/10.1101/2025.11.03.686234; this version posted November 4, 2025. The copyright holder for this preprint (which was not certified by peer review) is the author/funder, who has granted bioRxiv a license to display the preprint in perpetuity. It is made available under aCC-BY 4.0 International license.

Supplementary Figure 2. GSEA of remaining CoGAPS patterns

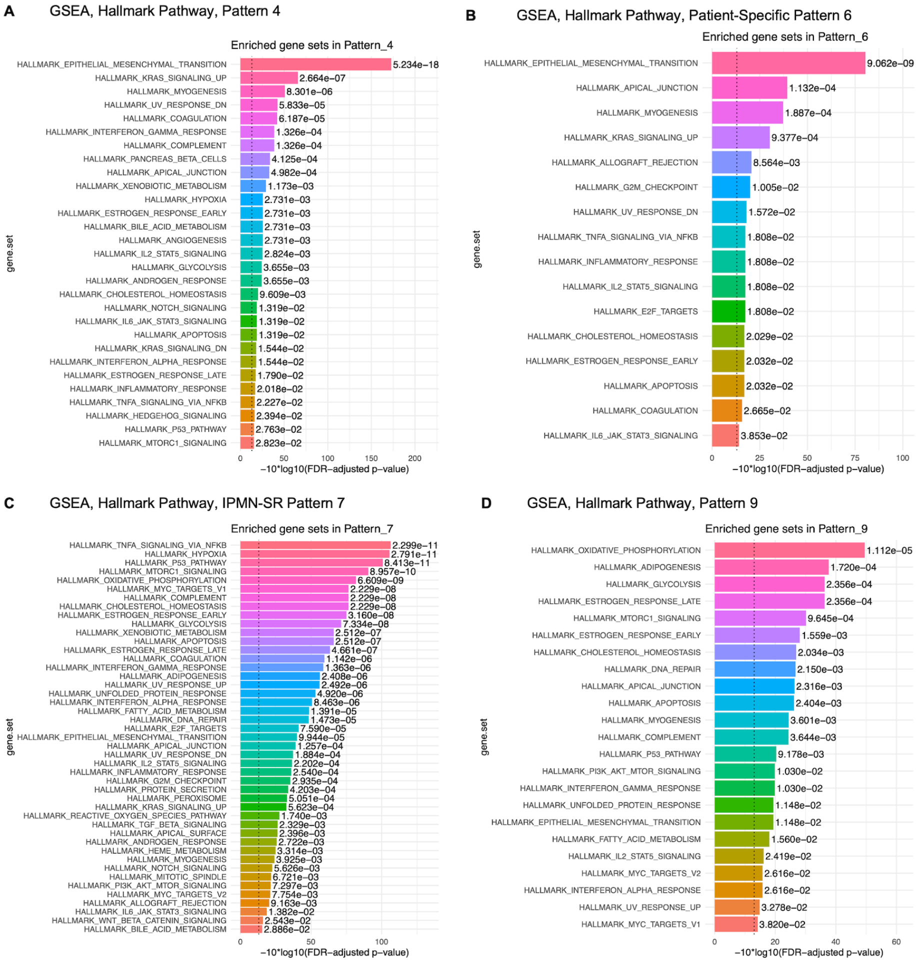

bioRxiv preprint doi: https://doi.org/10.1101/2025.11.03.686234; this version posted November 4, 2025. The copyright holder for this preprint (which was not certified by peer review) is the author/funder, who has granted bioRxiv a license to display the preprint in perpetuity. It is made available under aCC-BY 4.0 International license.

Supplementary Figure 2. GSEA of remaining CoGAPS patterns

E Pattern weights with WES information grouped by driver gene

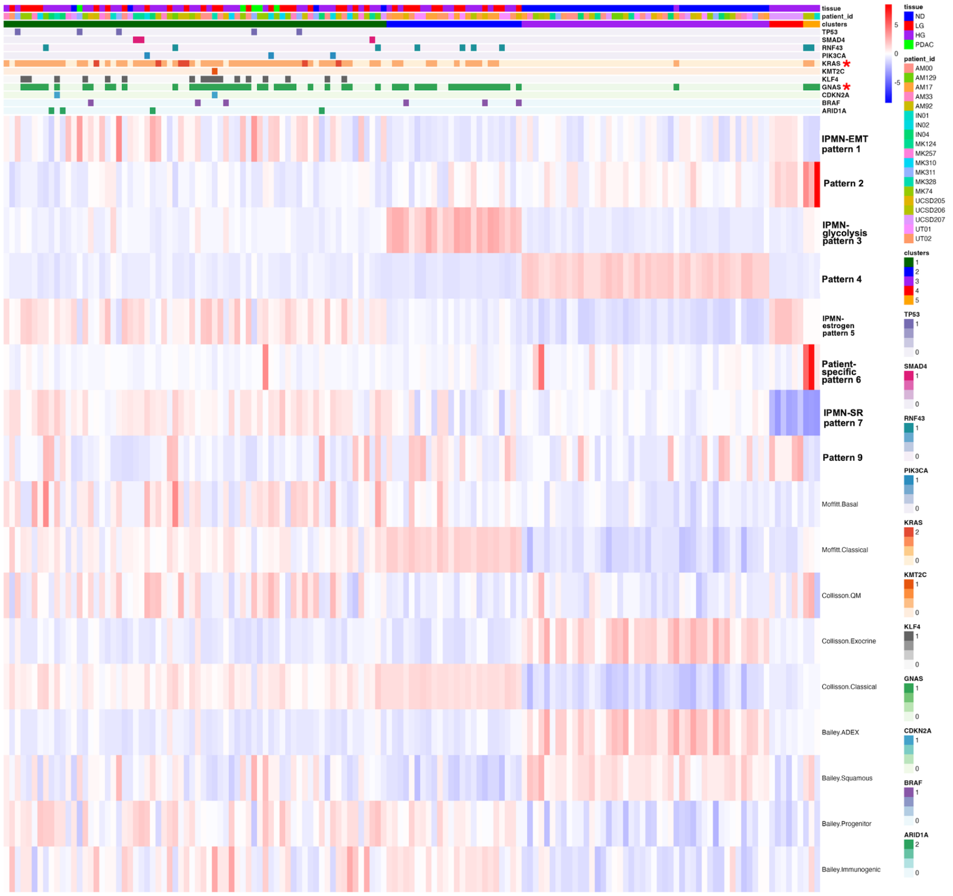

F Fibroblast fraction by cluster

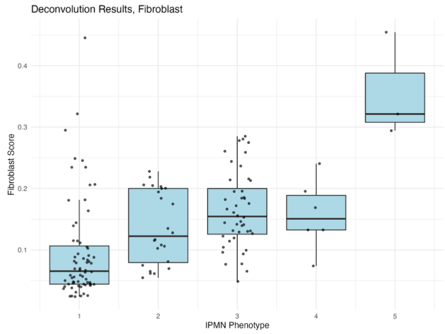

G Fibroblast fraction correlates with IPMN-EMT pattern 1 weight in IPMN

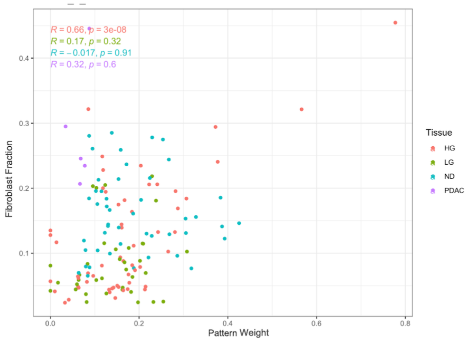

bioRxiv preprint doi: https://doi.org/10.1101/2025.11.03.686234; this version posted November 4, 2025. The copyright holder for this preprint (which was not certified by peer review) is the author/funder, who has granted bioRxiv a license to display the preprint in perpetuity. It is made available under aCC-BY 4.0 International license.

Supplementary Figure 3. IPMN cross-comparison

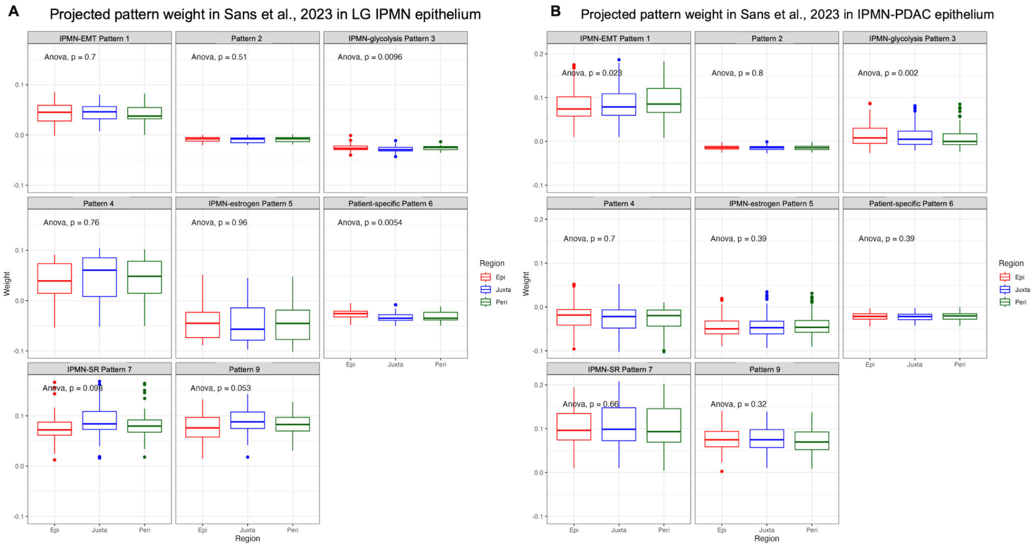

bioRxiv preprint doi: https://doi.org/10.1101/2025.11.03.686234; this version posted November 4, 2025. The copyright holder for this preprint (which was not certified by peer review) is the author/funder, who has granted bioRxiv a license to display the preprint in perpetuity. It is made available under aCC-BY 4.0 International license.

Supplementary Figure 4. IPMN hotspot analysis

A Percent of hotspots deemed as interactions between a given pattern and cell type, average across LG IPMN samples

	IPMN-EMT pattern 1	Pattern 2	IPMN-glycolysis pattern 3	Pattern 4	IPMN-estrogen pattern 5	Patient-specific pattern 6	IPMN-SR pattern 7	Pattern 9
B.activated	10.30	17.27	6.85	12.64	2.53	16.65	2.35	3.57
B.general	15.35	6.63	9.79	8.71	14.51	8.35	15.22	15.88
B.memory	10.00	10.19	6.13	11.93	6.30	7.87	7.14	9.50
B.naive	6.07	11.67	3.03	8.48	4.42	11.16	3.33	3.95
CD16.NK	9.84	11.95	9.52	5.74	10.23	15.74	5.35	5.83
CD56.NK	6.74	5.83	1.97	8.17	3.94	5.56	5.08	5.56
DC1	5.15	6.45	4.76	3.39	7.30	6.57	5.22	6.12
DC2.3	16.67	7.07	6.33	14.26	7.97	12.24	9.90	12.73
Effector.CD8.T	14.03	9.40	10.56	9.61	5.92	15.08	3.59	4.33
Endothelial	16.21	23.44	10.25	19.07	1.58	22.43	4.62	7.30
Epithelial.	12.02	5.42	17.41	9.34	29.79	0.79	36.54	32.54
Fibroblast	27.97	16.78	8.88	25.19	1.81	28.14	5.34	9.35
IFN.response.T	6.72	6.44	13.38	5.91	12.68	9.89	6.74	5.55
MAIT	8.61	9.58	7.61	9.71	6.48	10.76	6.79	6.96
Memory.CD4T	10.41	9.84	10.94	6.42	11.32	9.66	12.04	11.98
Memory.CD8T	10.17	6.59	5.88	15.97	4.23	7.06	10.87	10.21
Myeloid.gen	11.27	18.16	13.27	9.93	5.83	19.64	3.31	3.15
NK.T	10.34	15.67	14.34	7.79	10.09	15.83	3.20	3.68
NK.general	11.41	23.45	20.98	5.60	13.73	24.28	5.22	4.87
Naive.CD4.T	7.15	11.58	5.97	16.26	4.29	7.43	10.03	11.90
Naive.CD8.T	9.23	12.02	8.03	16.48	4.66	7.09	9.83	10.07
T.general	10.92	16.76	12.44	11.70	5.50	18.71	3.36	4.44
Treg	9.80	11.64	9.93	6.45	6.81	16.81	3.63	3.75
Trem	7.13	11.47	12.72	4.62	12.36	14.53	7.65	6.38
mac1	17.12	14.74	8.28	16.44	5.94	15.99	9.72	10.63
mac2	19.55	10.75	8.90	13.35	9.49	16.65	8.39	11.35
mast	20.57	10.72	9.80	16.52	8.88	14.64	12.85	14.05
mono1	11.75	11.98	4.89	13.42	6.31	16.14	3.41	7.44
mono2	18.62	14.82	9.14	16.36	3.33	22.18	1.83	4.96
pDC	8.89	14.52	5.77	15.91	3.87	10.27	7.90	8.24
plasma	16.62	10.64	9.63	13.60	11.18	14.67	12.91	14.85

bioRxiv preprint doi: https://doi.org/10.1101/2025.11.03.686234; this version posted November 4, 2025. The copyright holder for this preprint (which was not certified by peer review) is the author/funder, who has granted bioRxiv a license to display the preprint in perpetuity. It is made available under aCC-BY 4.0 International license.

Supplementary Figure 4. IPMN hotspot analysis

B Percent of hotspots deemed as interactions between a given pattern and cell type, average across HG IPMN samples

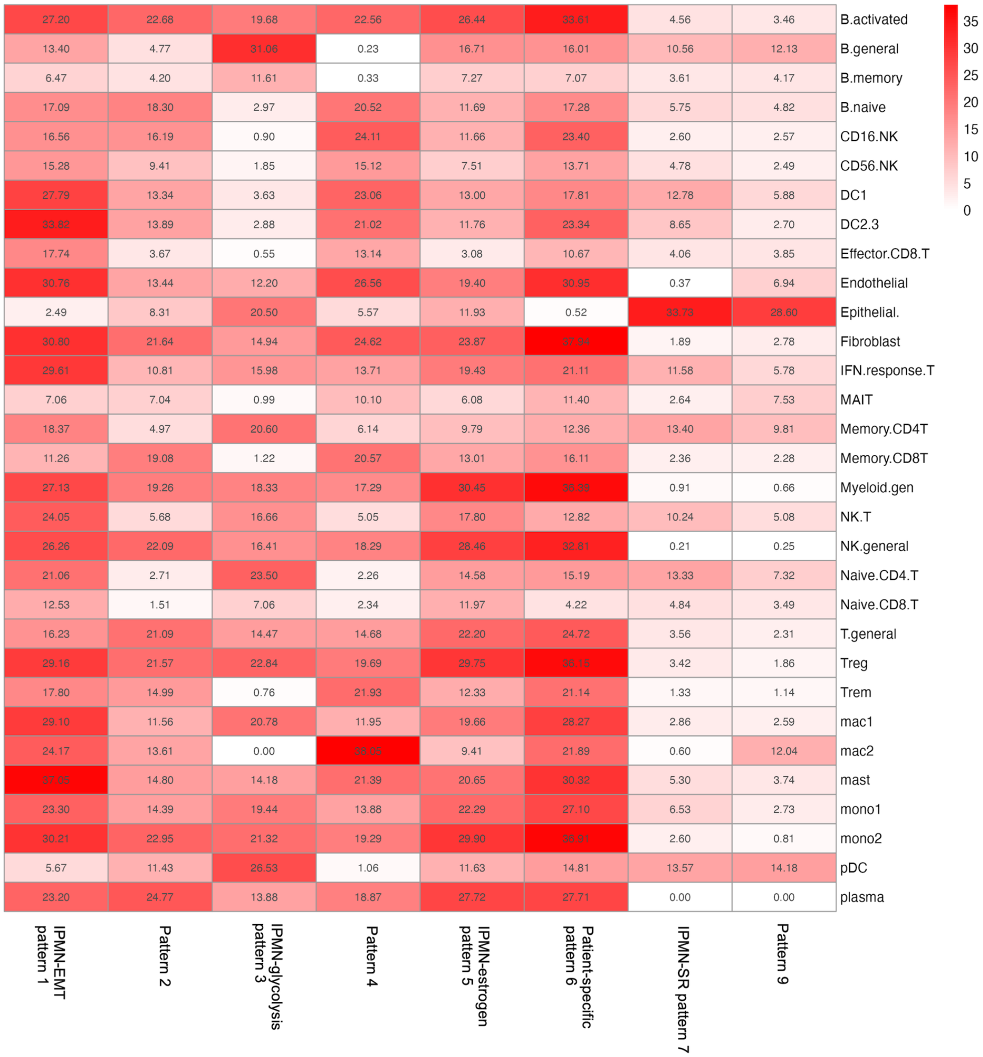

bioRxiv preprint doi: https://doi.org/10.1101/2025.11.03.686234; this version posted November 4, 2025. The copyright holder for this preprint (which was not certified by peer review) is the author/funder, who has granted bioRxiv a license to display the preprint in perpetuity. It is made available under aCC-BY 4.0 International license.

Supplementary Figure 4. IPMN hotspot analysis

C Percent of hotspots deemed as interactions between a given pattern and cell type, average across IPMN-PDAC samples

	IPMN-EMT pattern 1	Pattern 2	IPMN-glycolysis pattern 3	Pattern 4	IPMN-estrogen pattern 5	Patient-specific pattern 6	IPMN-SR pattern 7	Pattern 9
B.activated	10.15	15.20	5.05	14.55	7.01	14.69	5.73	3.98
B.general	10.26	11.11	4.18	10.28	4.68	15.02	7.05	9.51
B.memory	14.39	6.32	2.60	13.27	3.38	9.65	12.45	17.27
B.naive	6.60	5.16	0.04	14.15	4.12	7.18	6.28	14.77
CD16.NK	5.13	6.66	2.12	15.29	2.38	13.76	11.14	16.29
CD56.NK	11.18	5.58	1.55	19.66	0.95	11.90	10.03	20.80
DC1	9.57	6.92	3.15	18.28	6.84	13.92	10.88	15.62
DC2.3	15.34	3.31	6.02	16.70	1.81	6.57	16.67	16.13
Effector.CD8.T	3.03	10.64	1.85	15.89	8.95	10.65	6.13	12.48
Endothelial	9.99	11.81	3.95	16.30	4.75	18.31	7.02	12.57
Epithelial.	8.01	9.26	34.09	1.01	33.01	0.69	31.71	16.73
Fibroblast	13.52	25.40	6.10	28.76	5.26	33.47	0.37	4.32
IFN.response.T	15.19	2.61	5.70	6.29	5.30	6.95	8.58	11.66
MAIT	8.83	4.79	0.56	16.22	0.43	12.89	8.84	14.34
Memory.CD4T	4.19	8.48	7.99	3.30	6.56	7.76	5.61	4.80
Memory.CD8T	10.02	3.31	0.89	17.88	1.27	6.93	11.38	20.48
Myeloid.gen	13.77	21.04	7.73	10.93	8.41	17.82	3.07	3.15
NK.T	10.78	3.01	8.40	6.71	2.06	6.18	16.60	18.14
NK.general	13.09	26.51	17.62	5.30	15.14	18.37	4.27	1.35
Naive.CD4.T	9.96	3.12	1.20	15.79	1.85	6.21	13.28	18.34
Naive.CD8.T	7.63	7.08	1.05	9.50	6.07	10.09	7.53	10.63
T.general	4.63	13.20	4.73	11.09	4.57	12.99	3.60	4.44
Treg	10.04	6.37	4.46	10.79	0.96	11.00	14.76	18.26
Trem	6.20	21.96	6.60	1.12	11.57	9.67	3.50	1.92
mac1	18.61	8.60	18.82	4.62	10.07	5.41	19.08	13.48
mac2	23.79	5.00	5.68	13.41	3.04	13.18	14.77	16.90
mast	3.05	8.67	2.69	17.75	2.54	9.72	8.12	14.57
mono1	10.93	8.62	2.99	16.33	4.57	18.08	5.32	11.06
mono2	7.92	11.16	6.50	21.23	1.75	16.76	7.89	9.02
pDC	14.23	10.10	9.29	10.71	1.01	14.30	14.41	14.21
plasma	5.32	11.35	2.04	19.06	5.88	18.74	4.31	10.15

Color scale: 30, 25, 20, 15, 10, 5

bioRxiv preprint doi: https://doi.org/10.1101/2025.11.03.686234; this version posted November 4, 2025. The copyright holder for this preprint (which was not certified by peer review) is the author/funder, who has granted bioRxiv a license to display the preprint in perpetuity. It is made available under aCC-BY 4.0 International license.

Supplementary Figure 5. PanIN comparative analysis

A Spatial plots of cell type in Bell et al., 2024 PanIN samples

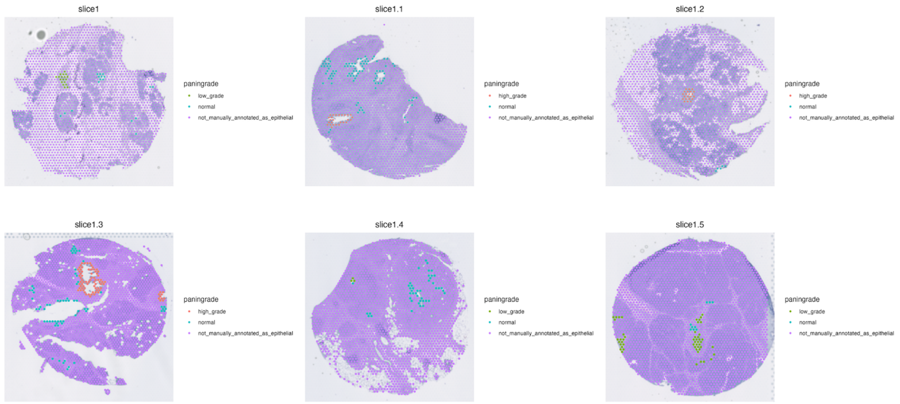

B Spatial plots of projected pattern weight in Bell et al., 2024 PanIN samples

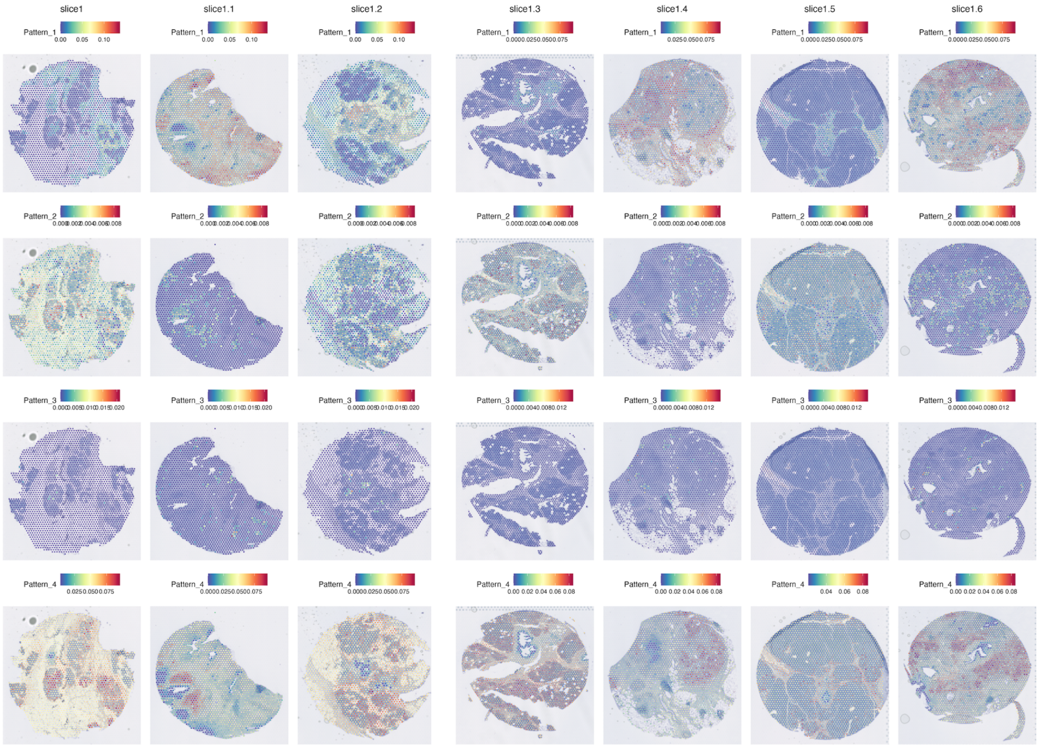

bioRxiv preprint doi: https://doi.org/10.1101/2025.11.03.686234; this version posted November 4, 2025. The copyright holder for this preprint (which was not certified by peer review) is the author/funder, who has granted bioRxiv a license to display the preprint in perpetuity. It is made available under aCC-BY 4.0 International license.

Supplementary Figure 5. PanIN comparative analysis

B Spatial plots of projected pattern weight in Bell et al., 2024 PanIN samples

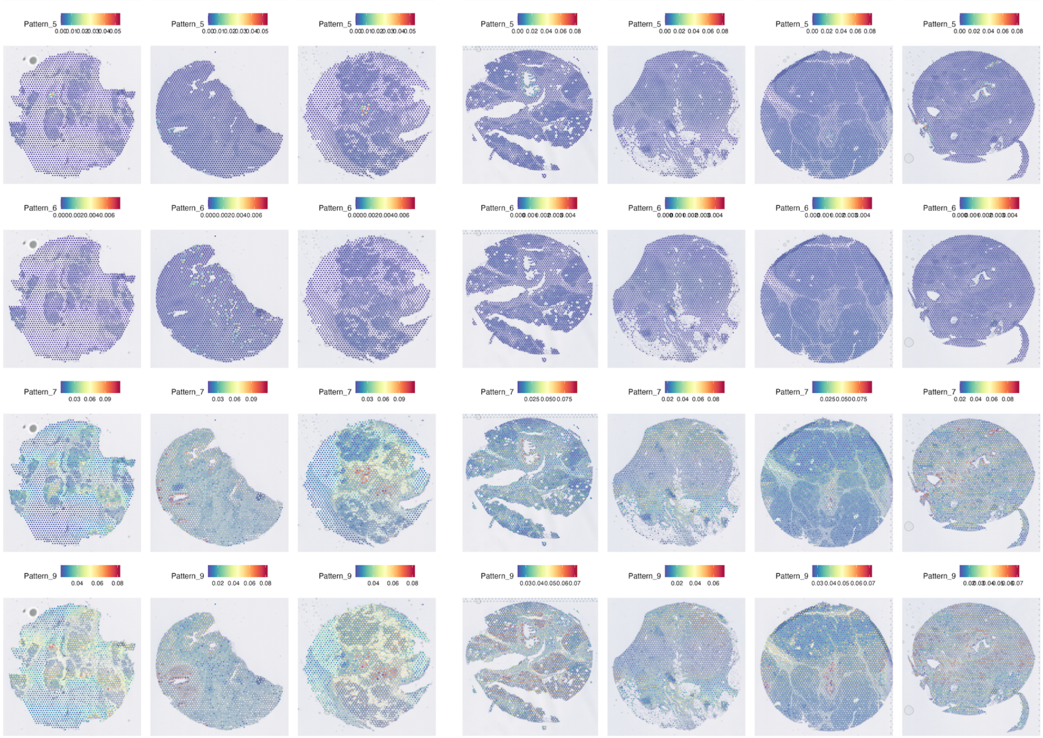

C Spatial plots of EMT-associated patterns and CAF scores in Bell et al., 2024 PanIN samples

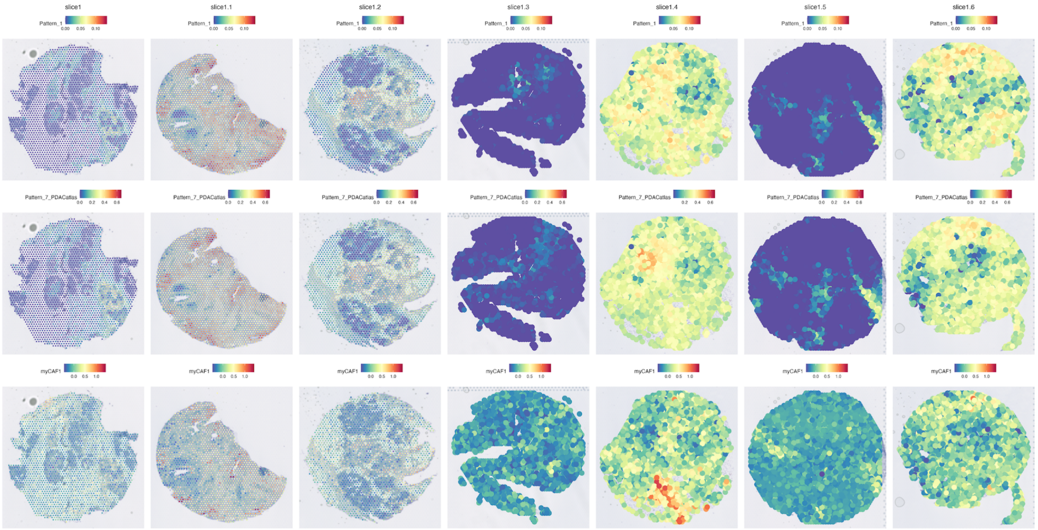

bioRxiv preprint doi: https://doi.org/10.1101/2025.11.03.686234; this version posted November 4, 2025. The copyright holder for this preprint (which was not certified by peer review) is the author/funder, who has granted bioRxiv a license to display the preprint in perpetuity. It is made available under aCC-BY 4.0 International license.

Supplementary Figure 5. PanIN comparative analysis

C Spatial plots of EMT-associated patterns and CAF scores in Bell et al., 2024 PanIN samples

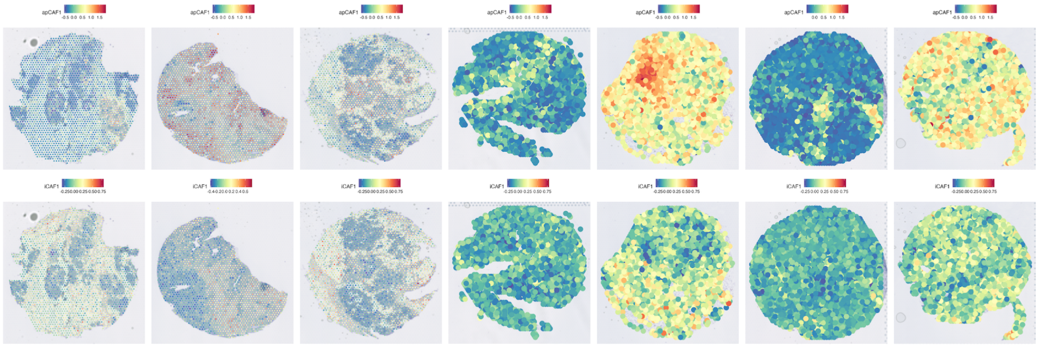

D Heat maps of hotspots of interaction between patterns and cell types

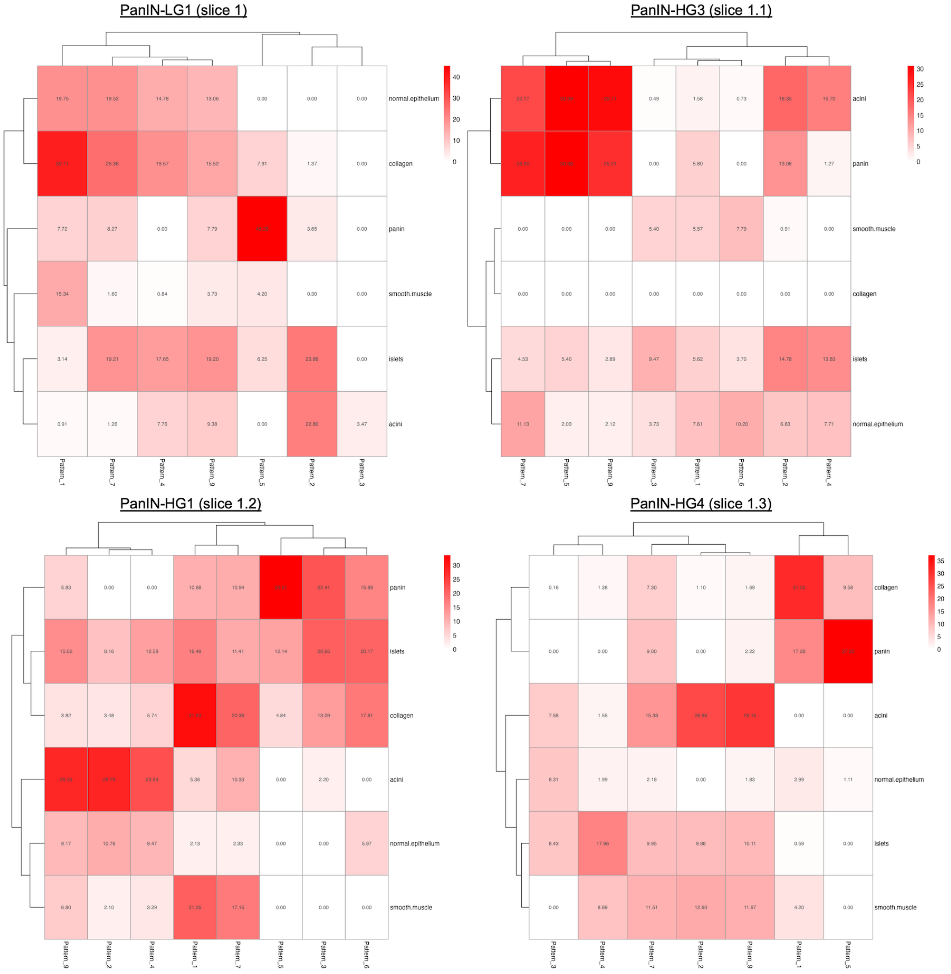

bioRxiv preprint doi: https://doi.org/10.1101/2025.11.03.686234; this version posted November 4, 2025. The copyright holder for this preprint (which was not certified by peer review) is the author/funder, who has granted bioRxiv a license to display the preprint in perpetuity. It is made available under aCC-BY 4.0 International license.

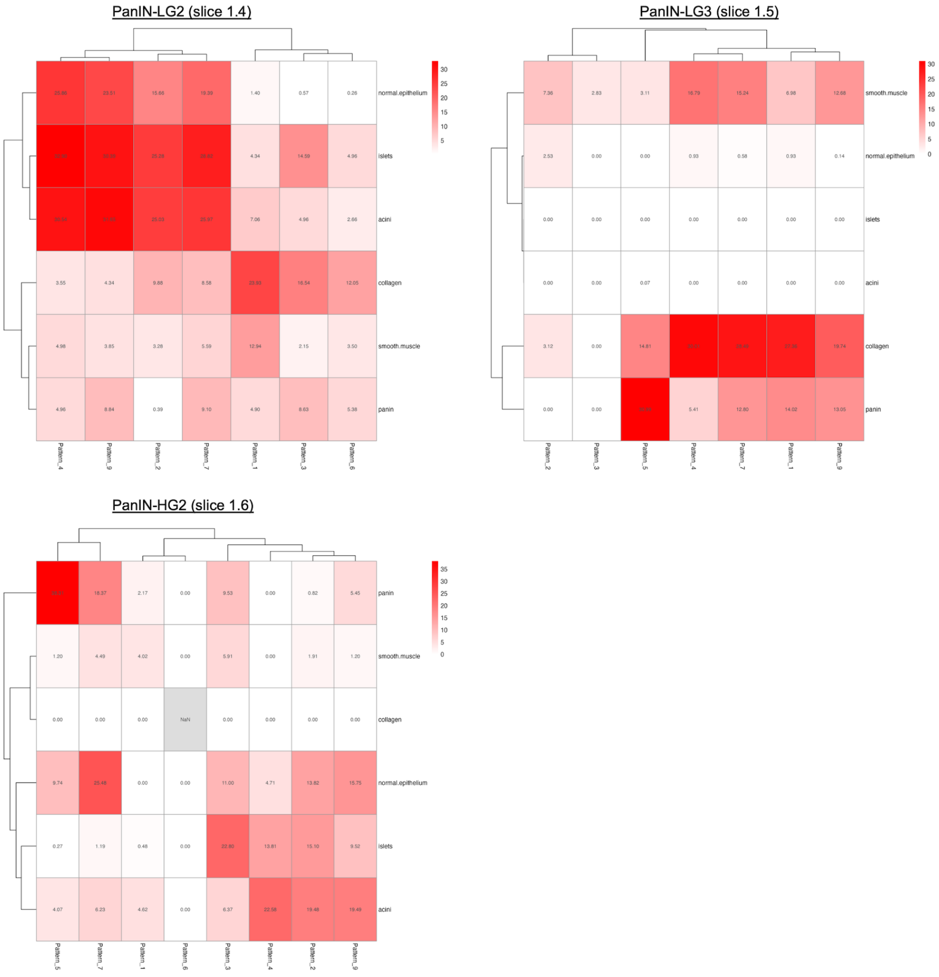

Supplement: 4 [file NIHPP2025.11.03.686234v1-supplement-4.pdf]
